# Identification of Discriminating Metabolic Pathways and Metabolites in Human PBMCs Stimulated by Various Pathogenic Agents

**DOI:** 10.3389/fphys.2018.00139

**Published:** 2018-02-27

**Authors:** Xiang Zhang, Adil Mardinoglu, Leo A. B. Joosten, Jan A. Kuivenhoven, Yang Li, Mihai G. Netea, Albert K. Groen

**Affiliations:** ^1^Department of Experimental Vascular Medicine, Academic Medical Center, University of Amsterdam, Amsterdam, Netherlands; ^2^Department of Chemical and Biological Engineering, Chalmers University of Technology, Gothenburg, Sweden; ^3^Science for Life Laboratory, KTH Royal Institute of Technology, Stockholm, Sweden; ^4^Department of Internal Medicine, Radboud University Nijmegen Medical Center, Nijmegen, Netherlands; ^5^Section Molecular Genetics, Department of Pediatrics, University Medical Center Groningen, University of Groningen, Groningen, Netherlands; ^6^Department of Genetics, University Medical Center Groningen, University of Groningen, Groningen, Netherlands; ^7^Department for Genomics & Immunoregulation, Life and Medical Sciences Institute, University of Bonn, Bonn, Germany; ^8^Department of Laboratory Medicine, University Medical Center Groningen, University of Groningen, Groningen, Netherlands

**Keywords:** innate immunity, metabolism, peripheral blood mononuclear cell, *Candida albicans*, lipopolysaccharides, *Mycobacterium tuberculosis*, *Borrelia burgdorferi*, genome scale metabolic model

## Abstract

Immunity and cellular metabolism are tightly interconnected but it is not clear whether different pathogens elicit specific metabolic responses. To address this issue, we studied differential metabolic regulation in peripheral blood mononuclear cells (PBMCs) of healthy volunteers challenged by *Candida albicans, Borrelia burgdorferi*, lipopolysaccharide, and *Mycobacterium tuberculosis in vitro*. By integrating gene expression data of stimulated PBMCs of healthy individuals with the KEGG pathways, we identified both common and pathogen-specific regulated pathways depending on the time of incubation. At 4 h of incubation, pathogenic agents inhibited expression of genes involved in both the glycolysis and oxidative phosphorylation pathways. In contrast, at 24 h of incubation, particularly glycolysis was enhanced while genes involved in oxidative phosphorylation remained unaltered in the PBMCs. In general, differential gene expression was less pronounced at 4 h compared to 24 h of incubation. KEGG pathway analysis allowed differentiation between effects induced by *Candida* and bacterial stimuli. Application of genome-scale metabolic model further generated a *Candida*-specific set of 103 reporter metabolites (e.g., desmosterol) that might serve as biomarkers discriminating *Candida*-stimulated PBMCs from bacteria-stimuated PBMCs. Our analysis also identified a set of 49 metabolites that allowed discrimination between the effects of *Borrelia burgdorferi*, lipopolysaccharide and *Mycobacterium tuberculosis*. We conclude that analysis of pathogen-induced effects on PBMCs by a combination of KEGG pathways and genome-scale metabolic model provides deep insight in the metabolic changes coupled to host defense.

## 1. Introduction

As the first line of host defense, the innate immune system can immediately sense and combat foreign pathogens (McGettrick and O'Neill, 2013; Mills and O'Neill, 2014). Cells of the innate immune system, such as monocytes and neutrophils recognize pathogens via pattern recognition receptors (PRRs) (McGettrick and O'Neill, 2013; Cheng et al., 2014; Mills and O'Neill, 2014). These PRRs, such as Toll-like receptors, NOD-like receptors, C-type lectin receptors, and RigI-helicases, are found on the plasma membrane of innate immune cells (McGettrick and O'Neill, 2013; Cheng et al., 2014; Mills and O'Neill, 2014). Activation of these PRRs leads to profound changes in gene expression and subsequent production of inflammatory mediators such as cytokines and chemokines (McGettrick and O'Neill, 2013; Pearce and Pearce, 2013; Cheng et al., 2014). Once innate immune cells are activated, they can trigger responses of the adaptive immune system (e.g., activate T lymphocytes) (Pearce et al., 2013; Mills and O'Neill, 2014).

Although often not realized, the responses of immune cells against pathogens are tightly linked to endogenous changes of metabolism (Mills and O'Neill, 2014). It is known that upon activation, immune cells (e.g., monocytes and T lymphocytes) dramatically shift from oxidative phosphorylation to aerobic glycolysis, in order to meet the rapidly increasing energy demand by processes such as cytokine production and cell proliferation (McGettrick and O'Neill, 2013; Pearce and Pearce, 2013; Pearce et al., 2013; Cheng et al., 2014). In addition, immune cells also increase the activity of the pentose phosphate pathway to provide sufficient nucleotide precursors for accelerated cell proliferation (e.g., T lymphocytes) (Pearce et al., 2013; Mills and O'Neill, 2014). Also, in lipopolysaccharide (LPS) challenged macrophages, succinate and citrate accumulate to regulate production of IL-1 β (Tannahill et al., 2013). Thus far, however, metabolism of activated immune cells has been mainly investigated after challenges with LPS, which only activates Toll-like receptor 4 (Bordbar et al., 2012; McGettrick and O'Neill, 2013; Tannahill et al., 2013). A recent study on the modulation of glycolysis and oxidative phosphorylation in immune cells stimulated with LPS and other TLR stimuli supported the concept that different stimuli may induce various metabolic programs in immune cells (Lachmandas et al., 2016).

To our knowledge, a comprehensive understanding of the metabolism of immune cells after stimulation of various PRRs (e.g., TLRs, NOD-like receptors—NLRs, C-type lectin receptors—CLRs, and RigI-helicases) has not yet been reported. In the current study, we interrogate which metabolic pathways and metabolites are altered upon activation by various pathogens. To this end, we systematically measured gene expression profiles in human peripheral blood mononuclear cells (PBMCs) stimulated by heat inactivated *Candida albicans* (*Candida*), *Borrelia burgdorferi* (*Borrelia*), *Escherichia coli*-derived LPS, and *Mycobacterium tuberculosis* (MTB). These four are typical stimuli of innate immune pathways. LPS is the prototypical stimulus recognized by TLR 4 (Ngkelo et al., 2012). *Candida* is recognized by TLRs and CLRs, and causes mucosal and systematic infection in immunocompromised individuals (Mayer et al., 2013). *Borrelia* is recognized by TLRs, NLRs, CLRs, and RigI-helicases and causes Lyme disease (Oosting et al., 2016). MTB is recognized by TLRs, NLRs, and CLRs and causes tuberculosis (Kleinnijenhuis et al., 2011).

To identify gene expression changes involved in metabolism, we ran Kyoto Encyclopedia of Genes and Genomes (KEGG) based metabolic pathway analysis and genome-scale metabolic model (GEM) based reporter metabolite analysis, respectively. KEGG pathway analyses are widely and successfully used in biomedical research over the last decade as a routine step of interpreting gene expression data (Kanehisa et al., 2012). As an alternative, genome scale metabolic models (GEMs) are increasingly used to interpret large-scale gene expression data sets. GEMs are represented by networks in which the nodes are metabolites and the connecting edges are metabolic reactions (Mardinoglu et al., 2013b; Bordbar et al., 2014). Generic human GEMs, such as Recon2 (Thiele et al., 2013) and HMR2 (Mardinoglu et al., 2014) represent our current knowledge of all established metabolic reactions involved in human energy metabolism and macromolecule biosynthesis. GEMs have mostly been used to identify key enzymes and metabolites that may serve as potential biomarkers and drug targets for non-alcoholic fatty liver disease, obesity, Alzheimer's disease, and cancer (Lewis et al., 2010; Mardinoglu et al., 2013a, 2014; Agren et al., 2014; Yizhak et al., 2014). Our analysis showed that KEGG pathway analysis allowed differentiation between effects induced by *Candida* and bacterial stimuli, and application of genome-scale metabolic model further generated a *Candida*-specific set of 103 reporter metabolites that might serve as biomarkers discriminating *Candida*-stimulated PBMCs from bacteria-stimulated PBMCs.

## 2. Materials and methods

### 2.1. Study populations

As described in the previous study (Smeekens et al., 2013), blood was collected after written informed consent from healthy volunteers. The study was approved by the Institutional Review Boards at Radboud University Nijmegen Medical Centre (RUNMC, Nijmegen, The Netherlands). The study was performed in accordance with the declaration of Helsinki. After informed consent was given, blood was collected by venipuncture into 10 ml EDTA syringes (Monoject, s-Hertogenbosch, The Netherlands).

### 2.2. Gene expression microarray data of stimulated PBMCs

As reported in in the previous study (Smeekens et al., 2013), we isolated PBMCs from healthy subjects by density centrifugation and stimulated them with heat-killed *C. albicans*(UC 820) (1 × 10^6^ per ml), heat-killed *B. burgdorferi, E. coli*-derived LPS (10 ng per ml), or heat-killed MTB (1 μg per ml), respectively for 4 or 24 h. PBMCs that were cultured in only RPMI medium were used as controls. Illumina Human HT-12 Expression BeadChips were used to measure gene expression levels at 4 and 24 h. Details about the experiment and processed data are available in GSE42606 archived by Gene Expression Omnibus.

### 2.3. Identification of differentially expressed genes

The raw gene expression data were preprocessed by using the lumi R package with default settings, which includes background correction, variance stabilizing transformation and quantile normalization (Lin et al., 2008). Principal component analysis was performed with the full gene expression data set by using the function prcomp in R. Valid paired samples were selected to perform differential expression analysis at 4 and 24 h separately. At 4 h, the size of paired samples for each stimulation were 19 (*Candida*), 25 (*Borrelia*), 19 (LPS), and 18 (MTB). At 24 h, the size of paired samples were 29 (*Candida*), 29 (*Borrelia*), 20 (LPS), and *N* = 30 (MTB). Illumina probe IDs were mapped to Ensembl gene IDs (Ensembl version 73) or Entrez gene IDs by using the lumiHumanIDMapping and biomaRt R packages (Durinck et al., 2009; Du et al., 2016). To exclude the influence of ambiguous probes (a probe ID corresponding to two or more gene IDs), only the probes that have unique gene IDs were used for differential gene expression analysis. Moreover, the hidden batch effect originated from microarray analysis were adjusted by applying surrogate variable analysis which is built in the sva R package (Leek and Storey, 2007, 2008; Leek et al., 2012). Gene expression levels of stimulated PBMCs were then compared to controls by using linear models and empirical Bayes statistics (Smyth, 2004). Both methods were implemented in the limma R package (Ritchie et al., 2015). Significance inference of differential expression was done with moderated t test (Ritchie et al., 2015) and the Benjamini-Hochberg procedure (Benjamini and Hochberg, 1995) was performed to calculate False Discovery Rate (FDR). In cases when a gene has multiple probes on the chip, the probe-level statistical test results were aggregated into a single gene-level statistic based on the smallest FDR.

### 2.4. Gene set enrichment analysis

In this study, the KEGG pathways and the generic human genome-scale metabolic model, HMR2 were used to analyze the gene expression data of human PBMCs stimulated by different pathogenic agents for 4 or 24 h. The KEGG pathway information was downloaded from the Molecular Signature Database v5.1 (Subramanian et al., 2005). There are in total 186 pathways and the related gene identifiers are Entrez gene IDs. Here we focused on 68 metabolic pathways since this study aims to identify metabolic signatures of stimulated human PBMCs. The HMR2 (SBML format) was downloaded from Human Metabolic Atlas (Pornputtapong et al., 2015). HMR2 contains 3,765 genes, 6,007 metabolites, and 8,181 reactions (Mardinoglu et al., 2014). Essentially, KEGG pathway analysis and reporter metabolite analysis are two gene set enrichment analysis methods. The difference between them is that KEGG pathway analysis uses protein constituted pathways to group genes, whereas reporter metabolite analysis uses metabolites to define gene sets. Since every metabolite serves as a gene set in reporter metabolite analysis, the information of which genes belonged to which metabolite was attained through using the piano R package (Väremo et al., 2013). The gene identifiers in HMR2 were annotated by Ensemble gene IDs (version 73). When KEGG pathways were used as gene sets, we computed average t statistics of pathways as the summary statistics:

(1) $$\begin{array}{r} Z_{pathway}=\frac{\overset{N_{pathway}}{\underset{i\;=\;1}{\sum }}t_{i}}{\sqrt{N_{pathway}}} \end{array}$$

This simple approach was first introduced by Irizarry et al. (2009). *Z*_*pathway*_ is the summary statistic of a pathway. *N*_*pathway*_ is the number of genes in the pathway and *t*_*i*_ is the modified t statistics of gene *i* in the pathway. When metabolites of HMR2 were translated to gene sets, the original reporter metabolite algorithm (Patil and Nielsen, 2005) was adapted to calculate summary statistics for metabolites. Patil and Nielsen (2005) defined reporter metabolites of which the expression levels were significantly changed. In the original reporter metabolite algorithm (Patil and Nielsen, 2005), the gene-level *P*-values were first converted to Z scores by using the inverse normal cumulative distribution. Then an aggregated Z score (gene set summary statistic) was calculated for each metabolite from the gene-level Z scores of its associated genes. Here we calculated summary statistics for metabolites directly with the gene-level modified t statistics:

(2) $$\begin{array}{r} Z_{metabolite}=\frac{\overset{N_{metabolite}}{\underset{i\;=\;1}{\sum }}t_{i}}{\sqrt{N_{metabolite}}} \end{array}$$

*Z*_*metabolite*_ is the summary statistics of a metabolite, and *t*_*i*_ is the t statistics of gene *i* associated with the metabolite. *N*_*metabolite*_ is the number of genes associated with the metabolite.

Regarding statistical inference, we calculated a *P*-value for each gene set based on its background distribution of summary statistics. However, unlike the original reporter metabolite algorithm (Patil and Nielsen, 2005), which derived background distributions by randomly sampling genes from the GEM, we applied sample permutations to derive such background distributions. Comparing gene/sample permutations is out of the scope of this manuscript. Goeman and Bühlmann (2007) extensively discussed this topic previously. The sample labels (stimulated or control) were randomly shuffled within each pair of samples (PBMCs derived from the same donor). As the next step, we repeated the same procedures as described previously to recalculate the gene-level as well as the summary statistics. In total, we performed such permutations 10,000 times for each stimulation case. The resulted permutation Z scores were used to represent the enrichment:

(3) $$\begin{array}{r} \text{Enrichment score}=\frac{Z-mean\left(Z_{null}\right)}{sd\left(Z_{null}\right)} \end{array}$$

*Z* is the summary statistic of a gene set (either *Z*_*pathway*_ or *Z*_*metabolite*_). *Z*_*null*_ refer to the summary statistics of that gene set based on the sample permutations.

Permutation *P*-values were then calculated by using the function permp in the statmod R package. The algorithm underlying the permp function was developed by Phipson and Smyth (2010). Since we tested a number of pathways or metabolites simultaneously, we performed the Benjamini-Hochberg procedure (Benjamini and Hochberg, 1995) to derive the FDR. When a metabolite had a FDR value below 0.05, we defined that particular metabolite as a reporter metabolite.

### 2.5. Identification of discriminating metabolic pathways and reporter metabolites

We were interested in metabolic pathways and metabolites that can discriminate *Candida*-stimulated PBMCs from *Borrelia*, LPS, and MTB-stimulated PBMCs. We were also interested in metabolic pathways and metabolites that can discriminate *Borrelia*, LPS, and MTB-stimulated PBMCs. To this end, we first compared gene set enrichment results across PBMCs stimulated by *Candida, Borrelia*, LPS, and MTB after treatment at 4 and 24 h. We compared the 4-h gene expression profile of PBMCs stimulated by *Candida, Borrelia*, LPS, and MTB to the paired RPMI-treated PBMCs. We did the same regarding the 24-h gene expression profile. When a pathway or a metabolite had a FDR value below 0.05 and a positive enrichment score, we labeled its transcriptional regulation as “Up.” When a pathway or a metabolite had a FDR value below 0.05 and a negative enrichment score, we marked its transcriptional regulation as “Down.” The remaining pathways and metabolites were then denoted as “N.S.,” meaning no significant transcriptional changes. In the following analysis, comparisons of pathways or metabolites in PBMCs stimulated by various pathogens were done based on their “Up,” “Down,” and “N.S.” patterns. The euclidean distance was calculated to quantify similarity between two metabolic pathway gene expression patterns. The ggdendro R package was used to produce the dendrogram and the cmdscale function of the stat R package was used to produce the multidimensional scaling plot. To identify metabolic pathways and metabolites that were differentially regulated in a specific bacterial stimulation at both 4 and 24 h, we also compared gene set enrichment results across PBMCs stimulated by *Borrelia*, LPS, and MTB. Considering difficulty of interpretation, HMR2 subsystems (equivalent to pathways), including “Isolated,” “Artificial reactions,” “Exchange reactions,” “Pool reactions,” “Miscellaneous,” “Other amino acid,” and “Blood group biosynthesis” were not included in the analysis. To simplify data visualization, all the transport subsystems were not included as well. If a metabolite could be mapped to multiple subsystems, all the subsystems were included in the final results.

To evaluate whether pathogen-specific metabolism corresponded to a specific immune response, we focused on innate immunity genes provided by the database innateDB (Breuer et al., 2013). According to the innateDB, there are 1,057 innate immune genes in human. Our microarray platform measured 850 of these innate immune genes. Similar to the procedures in pathway analysis, when an innate immune gene had a FDR value below 0.05 and a positive t statistic, we labeled its transcriptional regulation as “Up.” When an innate immune gene had a FDR value below 0.05 and a negative t statistic, we marked its transcriptional regulation as “Down.” The remaining innate immune genes were then denoted as “N.S.,” meaning no significant transcriptional changes. Again we performed the multidimensional scaling analysis.

## 3. Results

### 3.1. Transcriptional regulation in metabolic pathways of human PBMCs stimulated by various pathogenic challenges

**Figure 1:**
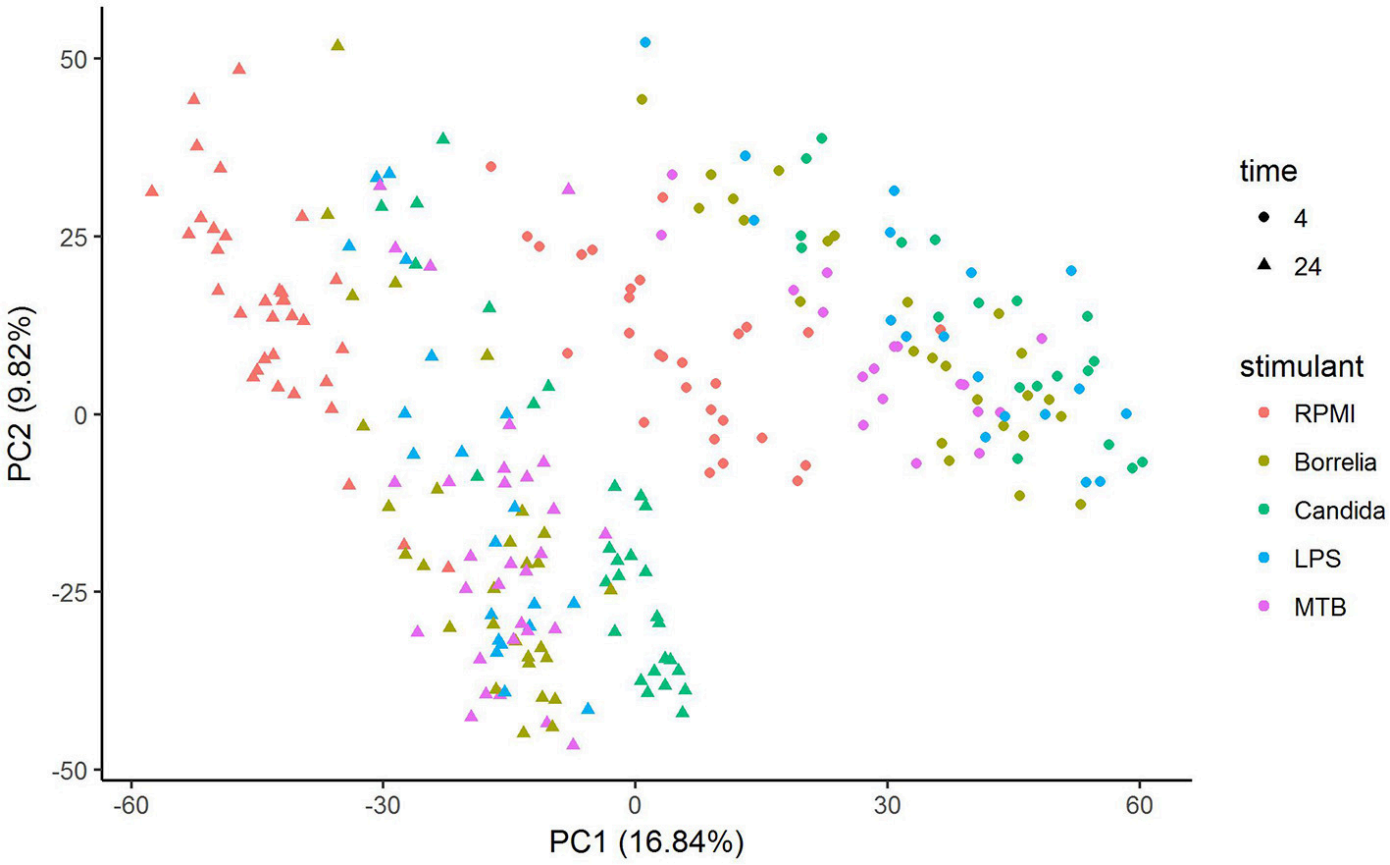
Principal component analysis of gene expression of human PBMCs stimulated by *Candida, Borrelia*, LPS and MTB for 4 and 24 h.

**Figure 2:**
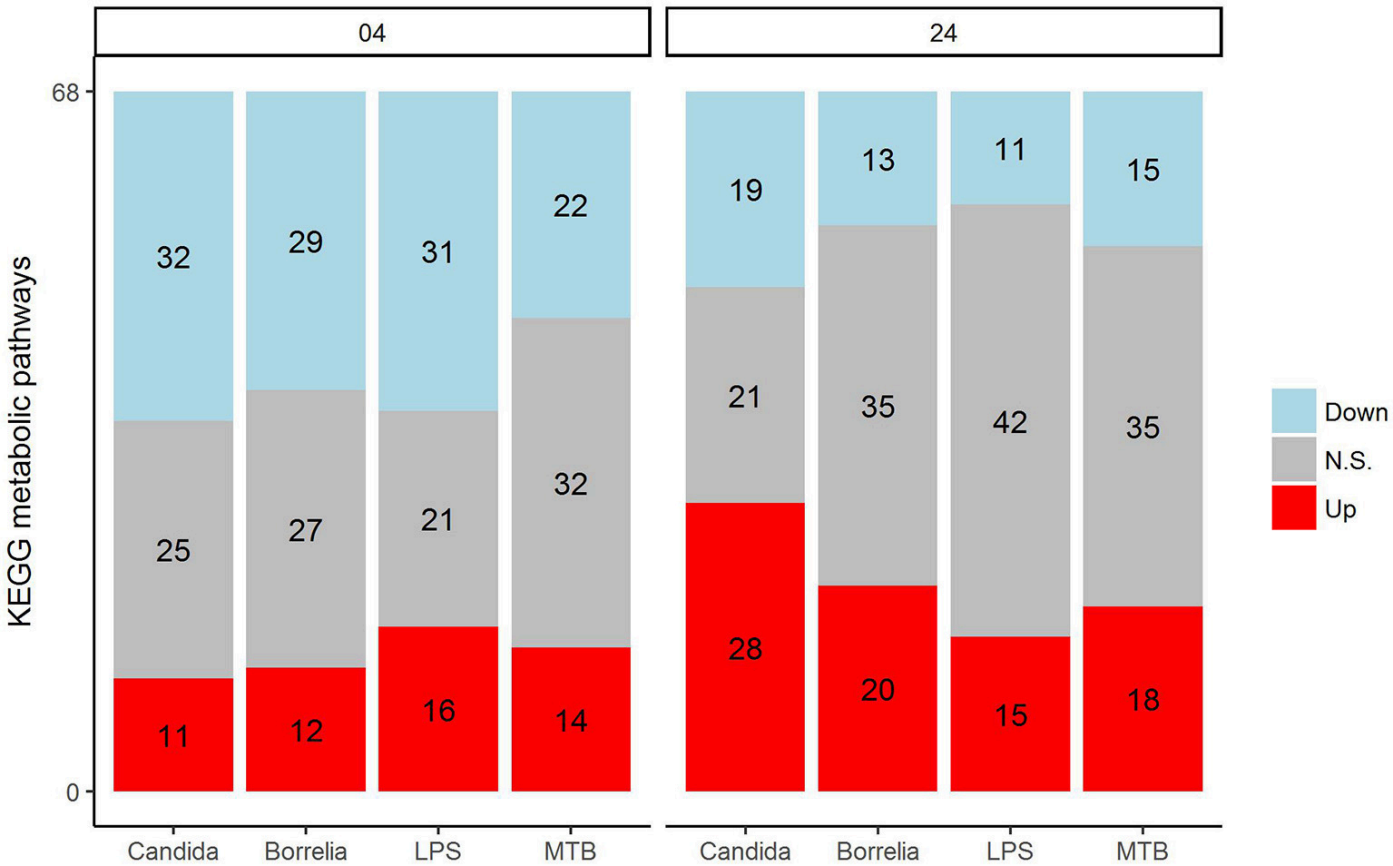
Distribution of significantly up-regulated (red), down-regulated (blue), and not significantly changed (gray) pathways in 68 KEGG metabolic pathways for *Candida, Borrelia*, LPS, and MTB-stimulated human PBMCs at 4 and 24 h. Any metabolic pathway is significantly changed if its FDR < 0.05.

**Figure 3:**
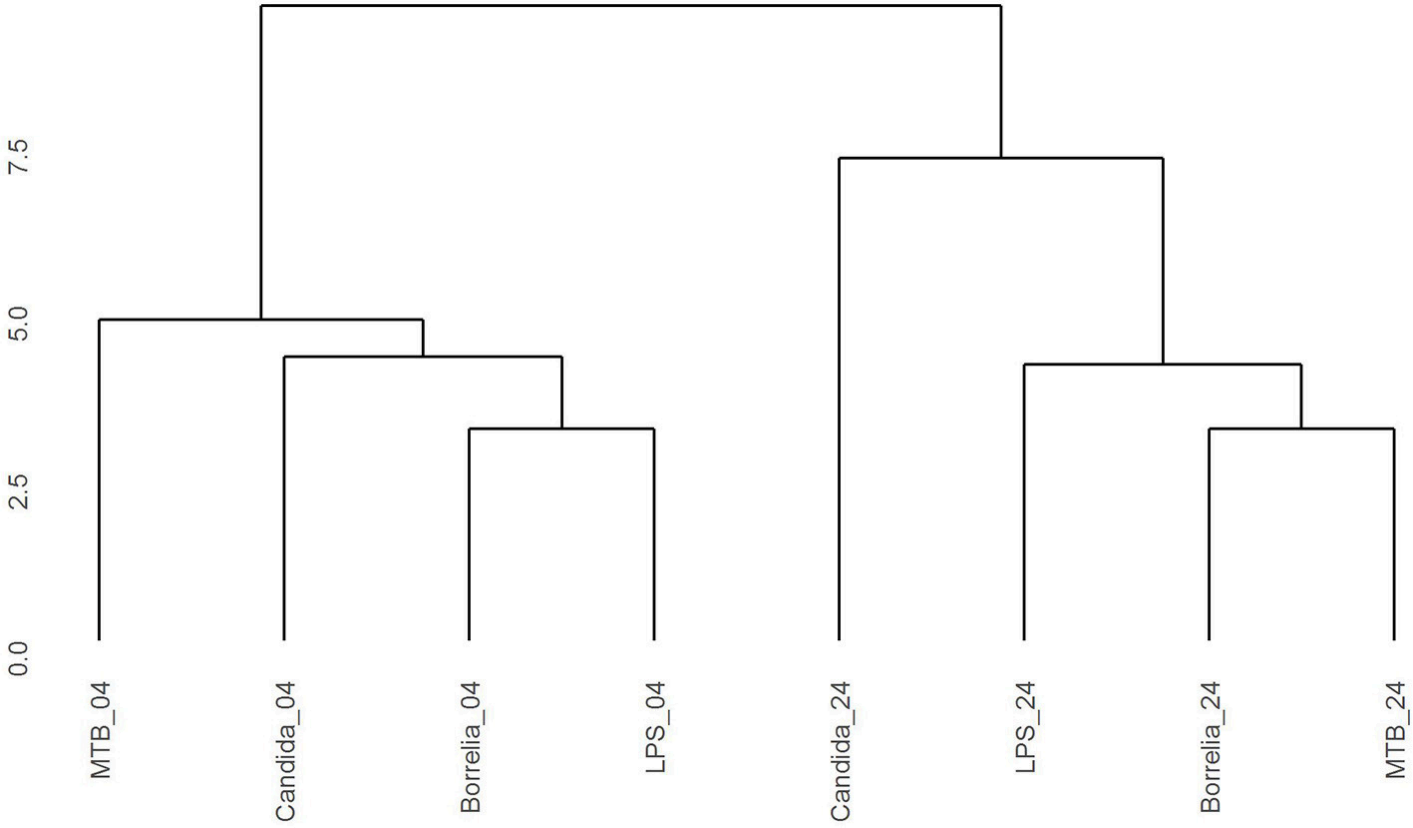
Hierarchical clustering gene expression pattern in KEGG metabolic pathways derived from human PBMCs stimulated by *Candida, Borrelia*, LPS, and MTB at 4 and 24 h. Euclidean distance is calculated to quantify similarity between two metabolic pathway gene expression pattern.

**Figure 4:**
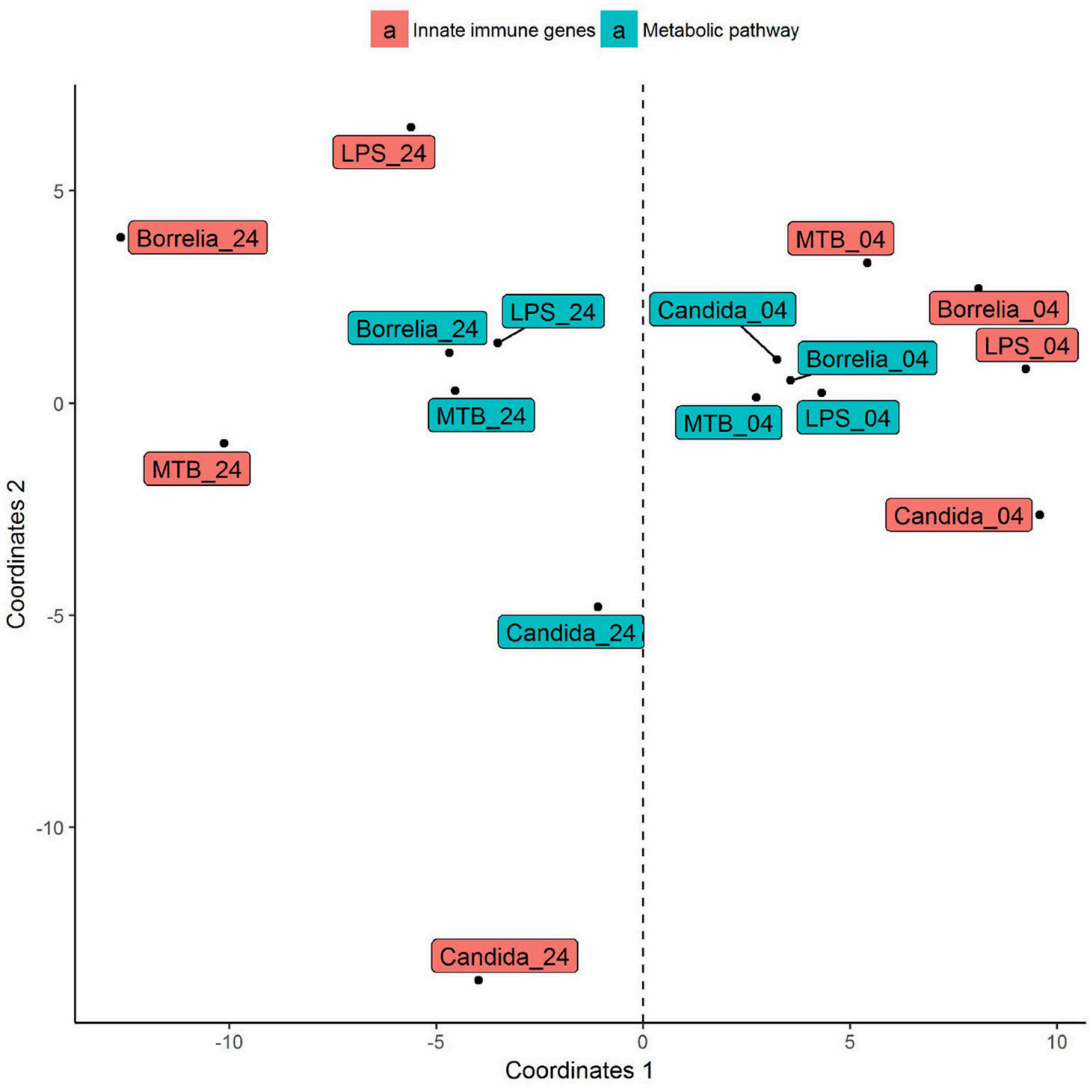
Multidimensional scaling of differential expression patterns of human PBMCs stimulated by *Candida, Borrelia*, LPS, and MTB at 4 and 24 h. Differential expression patterns were derived from genes involved in KEGG metabolic pathways and innate immunity. Euclidean distance is calculated to quantify similarity between two metabolic pathway gene expression pattern.

Depending on the duration and type of pathogenic stimulant, gene expression patterns of human PBMCs varied considerably. Along the axis of the first principal component, a clear separation of 4 and 24 h gene expression patterns was observed (Figure 1). To identify differentially regulated metabolic pathways in human PBMCs stimulated by heat-killed *Candida*, heat-killed *Borrelia*, LPS, and heat-killed MTB, we ran gene set enrichment analysis with KEGG metabolic pathways. In general, we observed more down than up-regulated metabolic pathways in stimulated PBMCs at 4 h. However, this was reversed at 24 h (Figure 2). Hierarchical cluster analysis revealed that metabolic pathway regulations were very different between 4 and 24 h irrespective of the stimuli used (Figure 3). Multidimensional scaling analysis confirmed the result of hierarchical clustering analysis. Furthermore, we observed that the clustering result based on metabolic pathways was consistent with the clustering outcome based on innate immunity genes at 24 h after stimulation (Figure 4).

### 3.2. Transcriptional regulation of energy metabolism in human PBMCs stimulated by various pathogenic challenges

At 4 h after stimulation, glycolysis pathway was down-regulated in *Candida* (Enrichment score = −5.88, FDR = 2.41 × 10^−4^), *Borrelia* (Enrichment score = −5.96, FDR = 3.09 × 10^−4^), LPS (Enrichment score = −5.83, FDR = 3.21 × 10^−4^), and MTB-stimulated (Enrichment score = −4.17. FDR = 0.0013) PBMCs. Oxidative phosphorylation pathway was also down-regulated in *Candida* (Enrichment score = −4.90, FDR = 2.41 × 10^−4^), *Borrelia* (Enrichment score = −4.60, FDR = 3.09 × 10^−4^), LPS (Enrichment score = −5.21, FDR = 3.21 × 10^−4^), and MTB-stimulated (Enrichment score = −3.82. FDR = 0.0013) PBMCs. At 24 h after stimulation, glycolysis pathway was up-regulated in *Candida* (Enrichment score = 4.33, FDR = 2.12 × 10^−4^), *Borrelia* (Enrichment score = 7.52, FDR = 3.09 × 10^−4^), LPS (Enrichment score = 2.99, FDR = 0.0019), and MTB-stimulated (Enrichment score = 7.51, FDR = 4.25 × 10^−4^) PBMCs. However, oxidative phosphorylation was not significantly changed in PBMCs stimulated by *Candida, Borrelia*, LPS, and MTB.

### 3.3. Discriminating metabolic pathways in human PBMCs stimulated by various pathogenic challenges

**Figure 5:**
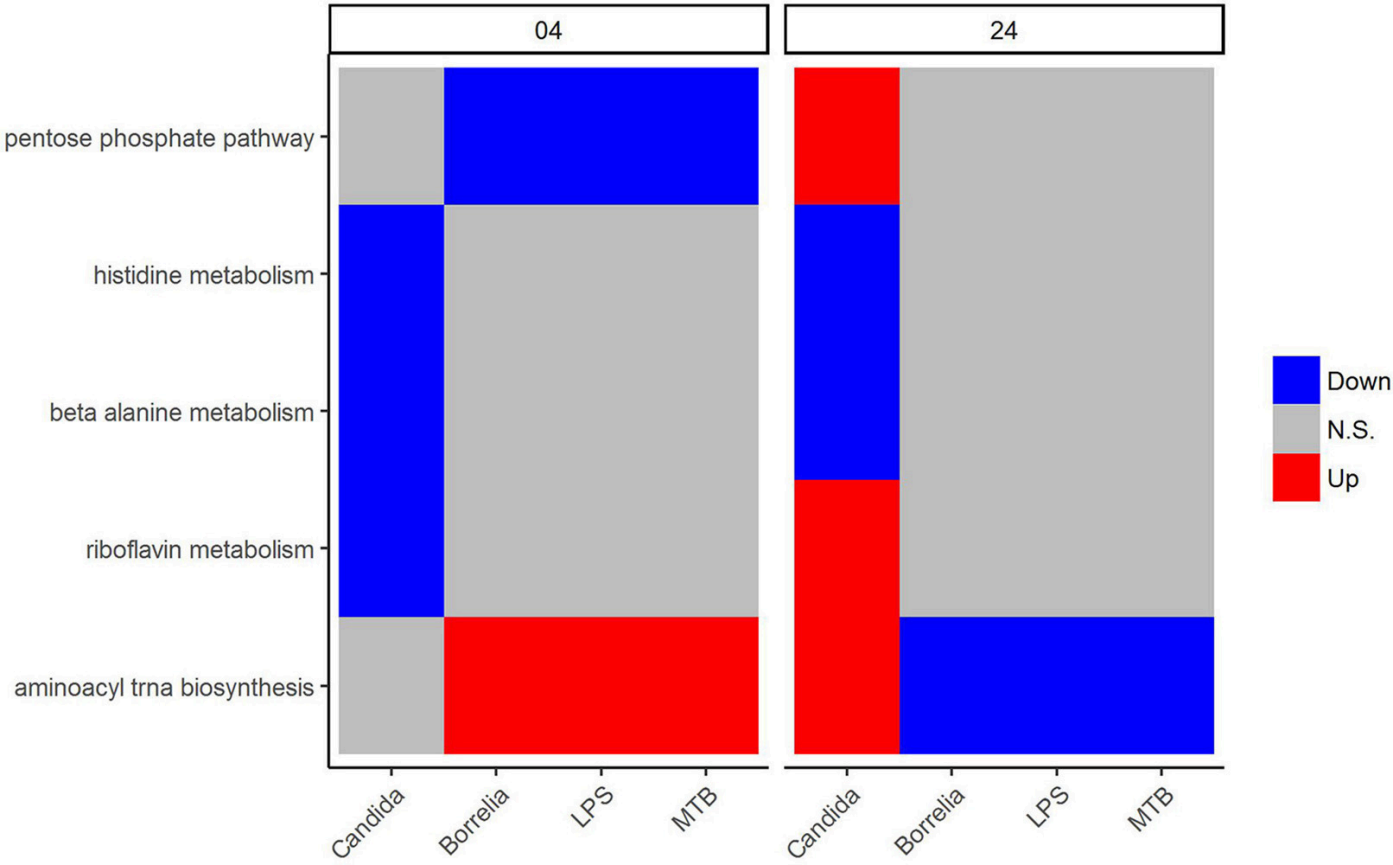
KEGG metabolic pathways that discriminated *Candida*-stimulated PBMCs from *Borrelia*, LPS, and MTB-stimulated human PBMCs. Blue refers to significantly down regulation. Red refers to significantly up regulation. Gray means not significantly changed. A pathway is significantly changed if its FDR < 0.05.

We focused on metabolic pathways that had the same transcriptional patterns in PBMCs stimulated by *Borrelia*, LPS, and MTB, but differed from *Candida*-stimulated PBMCs at both 4 and 24 h. The detail statistics for pathways were provided in the Supplementary Table 1. The pentose phosphate pathway was down-regulated in *Borrelia*, LPS, and MTB-stimulated PBMCs, but not in *Candida*-stimulated PBMCs at 4 h (Figure 5). However, at 24 h, the pentose phosphate pathway was up-regulated in *Candida*-stimulated PBMCs, but had no significant change in *Borrelia*, LPS, and MTB-stimulated PBMCs (Figure 5). Riboflavin, beta alanine and histidine metabolism were differentially regulated in *Candida*-stimulated PBMCs, but not significantly changed in *Borrelia*, LPS and MTB-stimulated PBMCs at both 4 and 24 h (Figure 5). Aminoacyl tRNA biosynthesis was up-regulated in *Borrelia*, LPS and MTB-stimulated PBMCs but not significantly changed in *Candida*-stimulated PBMCs at 4 h. However, this pathway was up-regulated in *Candida*-stimulated PBMCs but down-regulated in *Borrelia*, LPS, and MTB-stimulated PBMCs at 24 h (Figure 5).

**Figure 6:**
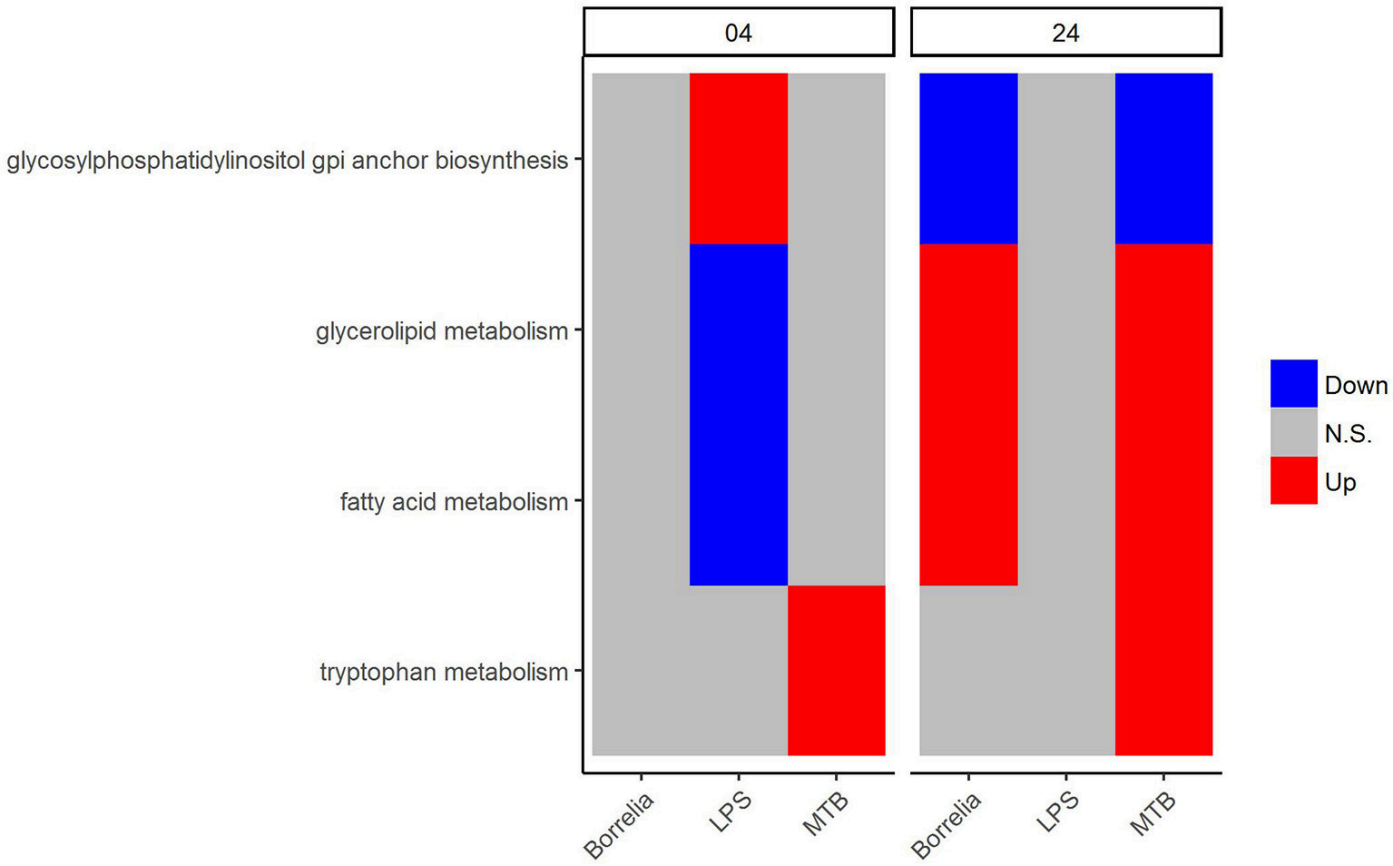
KEGG metabolic pathways that discriminated between *Borrelia*, LPS, and MTB-stimulated human PBMCs. Blue refers to significantly down regulation. Red refers to significantly up regulation. Gray means not significantly changed. A pathway is significantly changed if its FDR < 0.05.

Regarding the metabolic pathways that discriminated *Borrelia*, LPS and MTB-stimulated PBMCs, we observed that glycosylphosphatidylinositol GPI anchor biosynthesis was up-regulated in LPS-stimulated PBMCs but did not change in *Borrelia* and MTB-stimulated PBMCs at 4 h. However, at 24 h, this pathway was down-regulated in *Borrelia* and MTB-stimulated PBMCs whereas it remained unchanged in LPS-stimulated PBMCs (Figure 6). Similarly, fatty acid metabolism and glycerolipid metabolism were down-regulated in LPS-stimulated PBMCs but not in *Borrelia* and MTB-stimulated PBMCs at 4 h. This pathway was up-regulated in *Borrelia* and MTB-stimulated PBMCs but did not change in LPS-stimulated PBMCs at 24 h (Figure 6). Tryptophan metabolism was differentially regulated in MTB-stimulated PBMCs, but not significantly changed in *Borrelia* and LPS-stimulated PBMCs at both 4 and 24 h (Figure 6). We did not identify a metabolic pathway that can discriminate *Borrelia*-stimulated PBMCs from LPS and MTB-stimulated PBMCs.

### 3.4. Discriminating metabolites in human PBMCs stimulated by various pathogenic challenges

**Figure 7:**
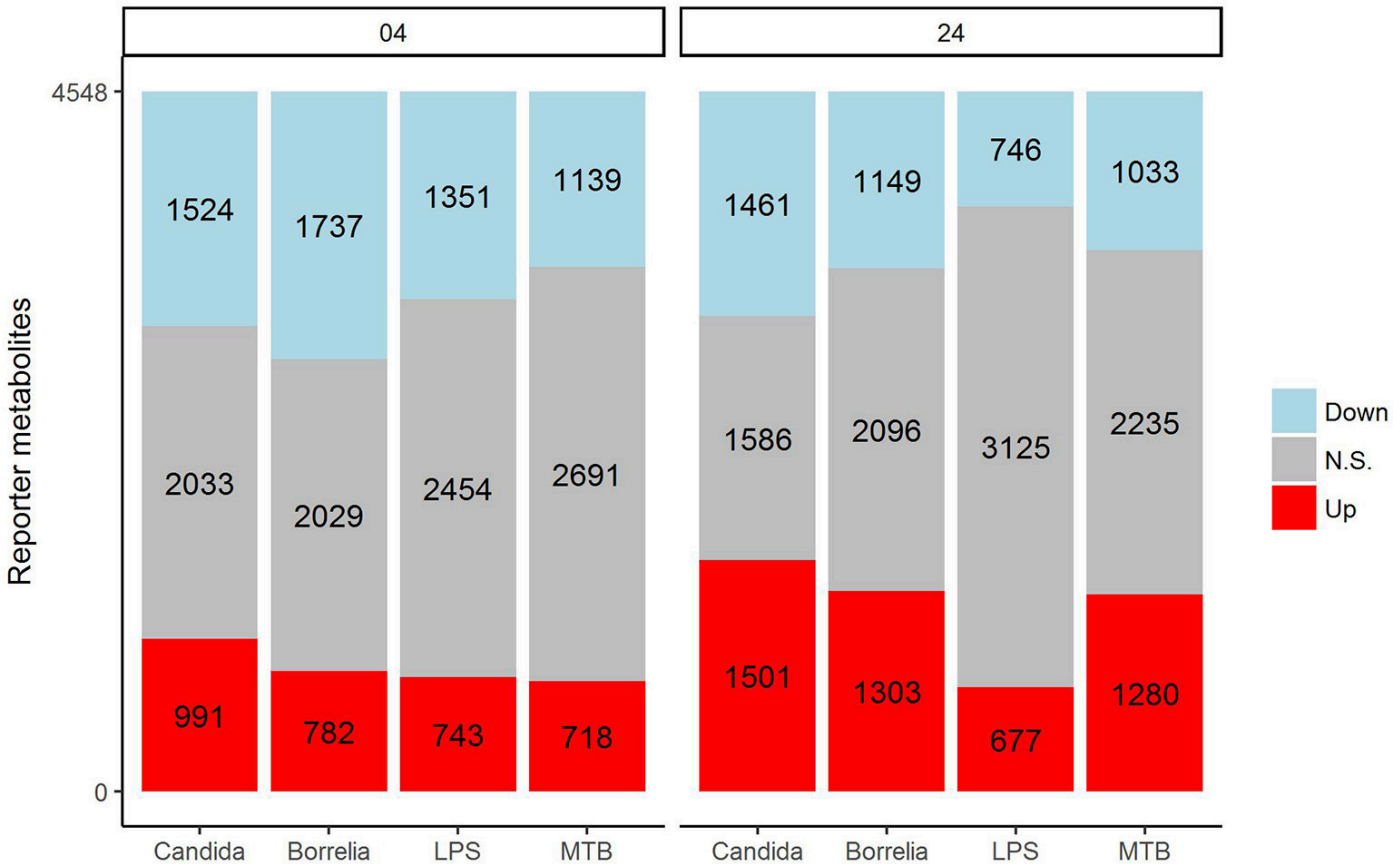
Distribution of significantly up-regulated (red), down-regulated (blue), and not significantly changed (gray) reporter metabolites for *Candida, Borrelia*, LPS, and MTB-stimulated human PBMCs at 4 and 24 h. When a reporter metabolite has FDR < 0.05, it is significant.

**Figure 8:**
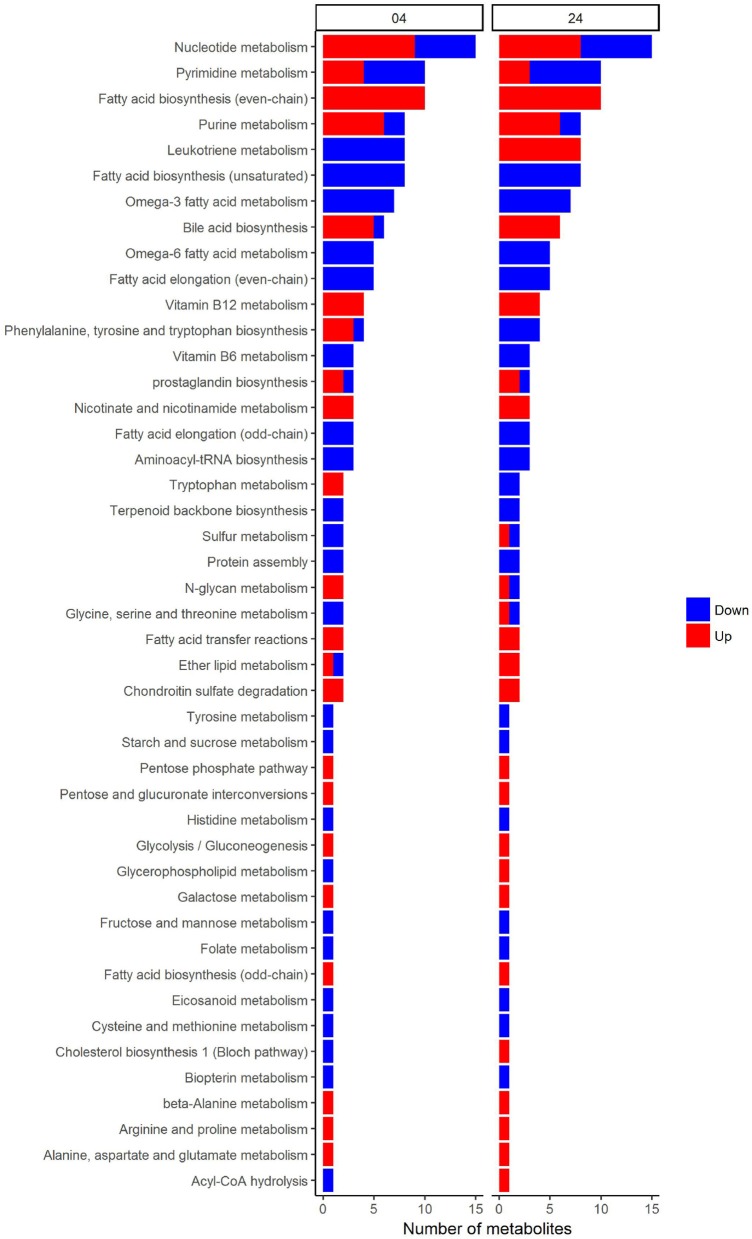
Reporter metabolites that discriminate *Candida*-stimulated PBMCs from *Borrelia*, LPS, and MTB-stimulated PBMCs at 4 and 24 h. These reporter metabolites were grouped based on their associated subsystems in HMR2. Blue denotes significant down-regulation. Red denotes significant up-regulation.

**Figure 9:**
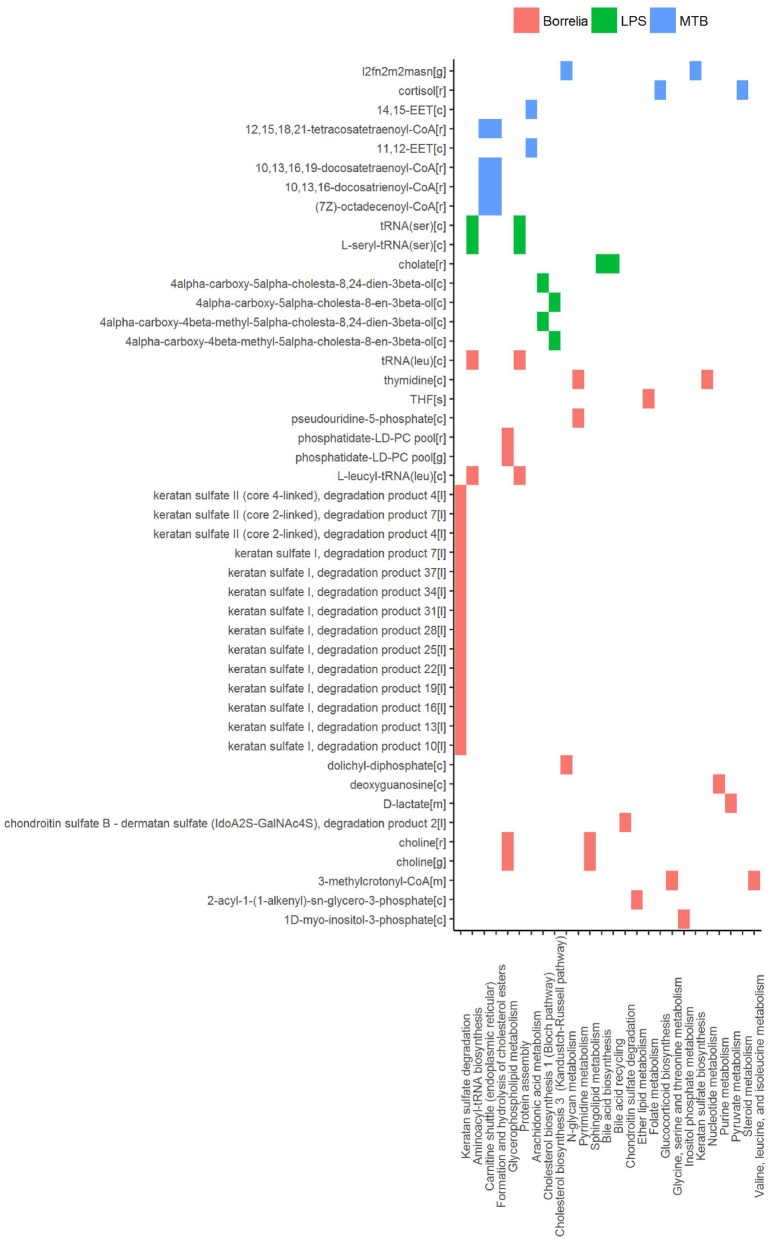
Reporter metabolites that discriminated *Borrelia*, LPS, and MTB-stimulated PBMCs at 4 and 24 h. Associated subsystems of these reporter metabolites are identified in HMR2.

In an attempt to identify metabolites that discriminated PBMCs with various stimuli, we ran reporter metabolite analysis with the human genome-scale metabolic model, HMR2. A total number of 4,548 metabolites were involved in the reporter metabolite analysis. We observed more down-regulated than up-regulated reporter metabolites in the stimulated PBMCs at 4 h. However, this pattern was reversed at 24 h (Figure 7). In a next step, we focused on reporter metabolites that were differentially regulated in *Candida*-stimulated PBMCs but not in PBMCs with bacterial stimuli at both 4 and 24 h. Among the identified reporter metabolites at 4 and 24 h, 103 of them were found specific for *Candida*-stimulated PBMCs. These 103 *Candida*-specific reporter metabolites participated in 45 pathways including nucleotide metabolism (15 reporter metabolites), and fatty acid biosynthesis (10 reporter metabolites; Figure 8). We also focused on reporter metabolites that can discriminate between *Borrelia*, LPS and MTB-stimulated PBMCs at both 4 and 24 h. We identified 32, 7, and 10 reporter metabolites that were specific for *Borrelia*, LPS and MTB-stimulated PBMCs, respectively (Figure 9). Statistics of all the pathogen-specific reporter metabolites were provided in Supplementary Table 2.

## 4. Discussion

The main finding of our study is that characterization of pathogen-dependent metabolic reprogramming in immune cells treated by various stimuli of innate immune pathway. For this purpose, we performed gene set enrichment analysis on gene expression data of human PBMCs treated with heat-killed *Candida*, heat-killed *Borrelia, E. coli*-derived LPS and heat-killed MTB. Either KEGG metabolic pathways or metabolites in human genome-scale metabolic models were used as gene sets. Our particular experimental setup with one fungal pathogen (*Candida*) and three bacterial inflammatory stimuli (*Borrelia*, LPS, and MTB) allowed us to identify metabolic signatures of *Candida*-induced host response, but also host response differences between bacterial challenges.

A very strong temporal effect on the expression of metabolic genes was observed. This observation is in line with the concept that stimulation period is a critical factor in immune response (Nagy and Haschemi, 2015; Hotamisligil, 2017). At 4 h after stimulation, both oxidative phosphorylation and glycolysis were down-regulated. At 24 h, however, gene expression of glycolysis showed up-regulation, whereas gene expression of oxidative phosphorylation remained unaltered in PBMCs. The observation of down-regulation of glycolysis genes after 4 h of stimulation is novel, and its impact for cell function warrants future studies. However, the observation at 24 h is consistent with literature data showing that activated immune cells shift toward glycolysis and away from oxidative phosphorylation (McGettrick and O'Neill, 2013; Pearce and Pearce, 2013; Pearce et al., 2013; Cheng et al., 2014).

For the purpose of identifying pathogen-dependent metabolic reprogramming in immune cells, we focused on metabolic pathways and metabolites that allow discrimination between various stimuli at both 4 and 24 h.

### 4.1. Five metabolic pathways can discriminate *Candida*-stimulated PBMCs from *borrelia*, LPS, and MTB-stimulated PBMCs

Five pathways, i.e., the pentose phosphate pathway, histidine metabolism, beta alanine metabolism, riboflavin metabolism, and aminoacyl tRNA biosynthesis, were identified to discriminate *Candida*-stimulated PBMCs from *Borrelia*, LPS, and MTB-stimulated PBMCs. Interestingly, we observed that the pentose phosphate pathway was differentially regulated in PBMCs stimulated by *Borrelia*, LPS, and MTB but not in *Candida*-stimulated PBMCs at 4 h. In contrast, at 24 h, this pathway was differentially regulated only in *Candida*-stimulated PBMCs but not significantly changed in *Borrelia*, LPS, and MTB-stimulated PBMCs. The pentose phosphate pathway was reported to support cytokine secretion in dendritic cells (Everts et al., 2014). Since cytokine production of human PBMCs depends on the type of stimulus (Henderson and Rippin, 1995), our observation of differential regulation in the pentose phosphate pathway likely indicates a specific function for *Candida* stimulated cytokine production. Indeed, our findings corroborate those of a recent study in which *Candida*-stimulated PBMCs were identified to have different cytokine profiles from bacteria-stimulated PBMCs (Li et al., 2016). On the other hand, little is known about the specific roles of the other four *Candida*-specific metabolic pathways in regulation of the immune response, and further investigation is warranted to validate these novel findings.

### 4.2. Four metabolic pathways can differentiate between *Borrelia*, LPS, and MTB-stimulated PBMCs

We further noted that three pathways (glycosylphosphatidylinositol GPI anchor biosynthesis, glycerolipid metabolism, fatty acid metabolism) discriminated LPS-stimulated PBMCs from *Borrelia* and MTB-stimulated PBMCs. Meanwhile, tryptophan metabolism differentiates MTB-stimulated PBMCs from *Borrelia* and LPS-stimulated PBMCs. We failed to identify pathways that allow discrimination *Borrelia*-stimulated PBMCs from LPS and MTB-stimulated PBMCs. Activation of tryptophan metabolism was previously reported in human marcophages *in vitro* upon MTB stimulation (Blumenthal et al., 2012), and a recent study (van Laarhoven et al., 2018) has identified a crucial role of tryptophan metabolism for the pathophysiology of tuberculous meningitis. In addition, enhancement of tryptophan catabolism is an IFN (interferon) γ-induced immune response in many different host cell types, and has been postulated to reduce the supply of tryptophan to bacterial pathogens (Moffett and Namboodiri, 2003; O'Neill et al., 2016). A reduced supply of tryptophan is linked to suppress T cell proliferation (Munn et al., 1999). Our observation of differential regulation of tryptophan in MTB-stimulated PBMCs might be related to different T cell proliferation after stimulation of MTB, compared to *Borrelia* and LPS.

### 4.3. Genome-scale metabolic model provides metabolic pathways with details

The KEGG pathway based analysis failed to identify metabolic pathways that discriminate *Borrelia*-stimulated PBMCs from LPS- and MTB-stimulated PBMCs. To explore potential differences in more depth, we ran the reporter metabolite analysis, which is a gene set enrichment analysis with a genome-scale metabolic model. A genome-scale metabolic model is comprised of metabolites and reactions between them. Compared to KEGG metabolic pathway information, the genome-scale metabolic model makes use of detailed information on biochemical reactions of pathways. For instance, for any enzyme catalyzing reaction, we can retrieve the genes encoding that enzyme in the genome-scale metabolic model. Moreover, metabolites can be products of some reactions and meanwhile act as substrates in other reactions. Consequently, reporter metabolite analysis based on genome-scale metabolic model does not repeat but complement results from KEGG pathway analysis. We used HMR2 in our analysis since we did not perform flux balance analysis.

### 4.4. 103 reporter metabolite can discriminate *Candida*-stimulated PBMCs from *Borrelia*, LPS, and MTB-stimulated PBMCs

In this study, we identified 103 reporter metabolites that were differentially regulated in *Candida*-stimulated PBMCs, but not in PBMCs stimulated with bacterial stimuli at both 4 and 24 h. A considerable number of these *Candida*-specific reporter metabolites were found to be related to lipid metabolism. The previous study (Smeekens et al., 2013) reported that *Candida* induced a type I IFN response that was distinct from *Borrelia*, LPS, and MTB stimulation. Interestingly, type I IFN was identified to influence *de novo* cholesterol biosynthesis and fatty acids biosynthesis in murine marcophages (York et al., 2015). Desmosterol, one of the *Candida*-specific reporter metabolites, is the last intermediary metabolite in the Bloch pathway of cholesterol biosynthesis. This metabolite was previously reported to coordinate cholesterol and fatty acid homeostasis, and affect anti-inflammatory function in macrophage (Spann et al., 2012). Taken together, we proposed that desmosterol might serve as a metabolic read out of the type I IFN response in *Candida*-stimulated PBMCs.

### 4.5. 49 reporter metabolites can discriminate between *Borrelia*, LPS, and MTB-stimulated PBMCs

In PBMCs stimulated by *Borrelia*, LPS, and MTB, 49 metabolites were identified to discriminate different kinds of pathogenic challenges. Within LPS-specific reporter metabolites, we observed intermediate metabolites present in the Bloch pathway and Kandutsch-Russell pathway (e.g., 4α-carboxy-5α-cholesta-8,24-dien-3β-ol). With mass spectrometry and isotope labeling techniques, (Mitsche et al., 2015) previously showed that different tissues or cell types were characterized by different flux distributions in the Bloch and Kandutsch-Russell pathway. Our observation indicates that there also might be condition-specific flux distribution in these two parallel cholesterol biosynthesis pathways. Within *MTB*-specific reporter metabolites, we observed two kind of epoxyeicosatrienoic acids, synthesized from arachidonic acid. Epoxyeicosatrienoic acids were reported to inhibit inflammatory gene expression in immune cells and animal models (Thomson et al., 2012).

## 5. Conclusions

In summary, by integrating gene expression data with KEGG metabolic pathways in combination with the human genome-scale metabolic model, a very sensitive method to characterize metabolic reprogramming in immune cells is obtained. Applying this methodology, we were able to discriminate metabolic pathways and metabolites in human PBMCs stimulated by *Candida, Borrelia*, LPS, and MTB. For instance, in the case of *Candida* we identified five differentially regulated pathways spanning metabolic regions from the pentose phosphate pathway to aminoacyl tRNA biosynthesis. Our analysis here, for the first time, provides insight into pathogen-specific metabolism which affects stimulus-dependent signal transduction and cytokine production in stimulated human PBMCs.

## Author contributions

XZ: Did the analysis and wrote the manuscript; LJ: Recruited the participants and performed stimulation experiment; AM, JK, YL, MN, and AG: Helped with manuscript writing.

### Conflict of interest statement

The authors declare that the research was conducted in the absence of any commercial or financial relationships that could be construed as a potential conflict of interest.
